# Life without a cell membrane: Challenging the specificity of bacterial endophytes within *Bryopsis *(Bryopsidales, Chlorophyta)

**DOI:** 10.1186/1471-2180-11-255

**Published:** 2011-11-21

**Authors:** Joke Hollants, Helen Decleyre, Frederik Leliaert, Olivier De Clerck, Anne Willems

**Affiliations:** 1 Laboratory of Microbiology, Department of Biochemistry and Microbiology, Ghent University, K.L. Ledeganckstraat 35, 9000 Ghent, Belgium; 2 Phycology Research Group, Department of Biology, Ghent University, Krijgslaan 281 (S8), 9000 Ghent, Belgium

## Abstract

**Background:**

The siphonous green macroalga *Bryopsis *has some remarkable characteristics. Besides hosting a rich endophytic bacterial flora, *Bryopsis *also displays extraordinary wound repair and propagation mechanisms. This latter feature includes the formation of protoplasts which can survive in the absence of a cell membrane for several minutes before regenerating into new individuals. This transient 'life without a membrane' state, however, challenges the specificity of the endophytic bacterial communities present and raises the question whether these bacteria are generalists, which are repeatedly acquired from the environment, or if there is some specificity towards the *Bryopsis *host.

**Results:**

To answer this question, we examined the temporal stability and the uniqueness of endobiotic bacterial communities within *Bryopsis *samples from the Mexican west coast after prolonged cultivation. DGGE analysis revealed that *Bryopsis *endophytic bacterial communities are rather stable and clearly distinct from the epiphytic and surrounding cultivation water bacterial communities. Although these endogenous communities consist of both facultative and obligate bacteria, results suggest that *Bryopsis *owns some intrinsic mechanisms to selectively maintain and/or attract specific bacteria after repeated wounding events in culture.

**Conclusions:**

This suggests that *Bryopsis *algae seem to master transient stages of life without a cell membrane well as they harbor specific - and possibly ecological significant - endophytic bacteria.

## Background

The marine green alga *Bryopsis *has long been suspected to harbor endogenous bacteria. These intracellular bacteria have been repeatedly observed in the cytoplasm as well as vacuolar regions of algal thalli and gametes by electron microscopy [[1,2] and personal observations see additional file 1], suggesting the presence of bacterial endophytes within *Bryopsis *is a natural phenomenon. Recently, the first insights were provided into the identity and diversity of these bacterial endophytes within two *Bryopsis *species from the Pacific Mexican coast [3]. Full length 16S rRNA gene analysis showed that the *Bryopsis *endophytic bacterial communities are quite low in diversity (i.e. only 7 bacterial OTUs detected) but taxonomically wide-ranging with the presence of *Arcobacter*, Bacteroidetes, Flavobacteriaceae, *Mycoplasma, Labrenzia*, Phyllobacteriaceae and Xanthomonadaceae species. Moreover, the same Bacteroidetes, *Mycoplasma*, Phyllobacteriaceae, and in particular Flavobacteriaceae bacteria, were detected in several *Bryopsis *samples collected hundreds of kilometers apart. This apparent spatial stability of the *Bryopsis*-bacterial endobiosis, however, raises the question whether these endophytes are a subset of the free-living bacterial community or whether there is some specificity towards the *Bryopsis *host. Although the distinctiveness between free-living and macroalgal-associated bacterial communities is well established [4-8], the extraordinary morphological and physiological characteristics of the *Bryopsis *host must have implications for the specificity of its bacterial endophytes. *Bryopsis *is a marine siphonous macroalga composed of a single, tubular shaped cell which contains multiple nuclei and chloroplasts in a thin cytoplasmic layer surrounding a large central vacuole [9]. While an organism composed of a giant, single cell would be prone to damage, siphonous macroalgae possess an intricate defense network that operates at various levels [7,10]. In *Bryopsis*, for example, the metabolite kahalalide F, which shows *in vitro *therapeutic activities, protects the alga from fish predation [11]. Even if damage does occur, a complex, multistep wound response is triggered [10,12] to which *Bryopsis *algae add a surprisingly feature, i.e. the formation of protoplasts [13]. These protoplasts are membraneless structures that can survive in seawater for 10-20 minutes. Subsequently, membranes and a cell wall are synthesized de novo surrounding each protoplast, which then develop into new *Bryopsis *plants. This not only suggests *Bryopsis *can exist - at least transiently -without a cell membrane, it also questions the nature of the association between the algal host and the endophytic bacterial communities present. Are these bacteria *Bryopsis*-specific, obligate endophytes (specialists) or are they rather generalists (facultative endogenous bacteria) which are repeatedly acquired from the local environment (epiphytic communities and/or surrounding sea water)?

To address this issue, we evaluated the temporal stability of the endobiotic bacterial communities after prolonged cultivation of *Bryopsis *isolates. We also examined the diversity of the epiphytic and surrounding water bacterial communities of five *Bryopsis *isolates in culture using the DGGE technique and subsequently compared these DGGE profiles with previously obtained DGGE banding patterns of *Bryopsis *endophytic bacterial communities [3].

## Methods

### Sample collection and DNA extraction

*Bryopsis hypnoides *(MX19 and MX263) and *Bryopsis pennata *var. *leprieurii *individuals (MX90, MX164 and MX344) were collected in February 2009 at five different sites along the Mexican west coast [3]. Living algal samples were transferred to the laboratory and unialgal *Bryopsis *cultures were formed by repeatedly isolating clean apical fragments. To preserve these unialgal cultures, apical fragments were monthly transferred to fresh sterile 1 × modified Provasoli enriched seawater [14]. All unialgal *Bryopsis *cultures were maintained in the laboratory at 23°C under a 12 h:12 h light/dark cycle with light intensities of 25-30 μE m^-2^s^-1^.

One year after the first endophytic community screening [3], all five *Bryopsis *MX samples were resubmitted to a total surface sterilization [15] and DNA extraction [16] in October 2010 to evaluate the temporal stability of the endophytic bacterial communities after prolonged cultivation. To address the specificity of the *Bryopsis-*bacterial endobiosis in culture, 50 ml of 30 day old cultivation water was collected from each *Bryopsis *MX culture that had been cultivated for two years (i.e. in February 2011). These cultivation water samples were serially filtered over a syringe filter holder with sterile 11 μm and 0.2 μm cellulose acetate filters (Sartorius Stedim Biotech GmbH, Germany) to remove small *Bryopsis *fragments and to retain the planktonic microbial fraction, respectively. Bacterial DNA was extracted from the 0.2 μm filters using the bead-beating method followed by phenol extraction and ethanol precipitation as described by Zwart *et al*. [17]. Parallel with these cultivation water samples, washing water samples were obtained from all five MX isolates by repeatedly vortexing the algae in 50 ml sterile artificial seawater (ASW). These washing water samples, containing the loosely *Bryopsis-*associated bacterial fraction, were processed as described above. Subsequently, approximately 1 gram of each washed *Bryopsis *MX sample was placed in 500 μl cetyltrimethylammonium bromide (CTAB) lysis buffer supplemented with 20 mg.mL-^1 ^proteinase K and 2.5 μl filter-sterilized Umonium Master (Huckert's International, Belgium) to eliminate the epiphytic bacterial fraction from the *Bryopsis *surface [15]. Samples were incubated for 30 minutes at 60°C and subsequently vortexed in 500 μl sterile ASW for 2 minutes. Algal material was removed by centrifugation and the supernatants' DNA originated from the epiphytic bacterial fraction was extracted using a CTAB protocol modified from Doyle and Doyle [16].

### DGGE and sequence analysis

The endophytic (EN-2010), epiphytic (EP), washing water (WW) and cultivation water (CW) bacterial community extracts were subjected to a nested-PCR DGGE approach. First, full length 16S rRNA gene amplification was carried out with the universal bacterial primers 27F/1492R following the protocol outlined in Lane [18]. PCR amplicons were purified using a Nucleofast 96 PCR clean up membrane system (Machery-Nagel, Germany) according to the manufacturer's instructions and subsequently submitted to a second PCR with primer pair F357-GC/R518 targeting the V3 region of the 16S rRNA gene. The latter amplification reaction and subsequent DGGE analysis were carried out as previously described [15], with a denaturing gradient of 45-65%. DGGE banding patterns were normalized using BioNumerics 5.1 software (Applied Maths, Belgium). As standard, a marker containing the V3 16S rRNA gene fragments of all bacterial endophyte and chloroplast OTUs formerly obtained from the five *Bryopsis *MX samples [3] was used (see additional file 2). The temporal stability of the endophytic communities was explored by visually comparing the normalized endophytic community profiles of MX sample's DNA extracts made in October 2009 (EN-2009) versus October 2010 (EN-2010). To study the specificity of the *Bryopsis-*bacterial endobiosis, normalized EP, WW and CW bacterial community profiles of each *Bryopsis *sample were comparatively clustered with previously obtained endophytic (EN-2009) DGGE banding patterns [15] using Dice similarity coefficients. A dendrogram was composed using the Unweighted Pair Group Method with Arithmetic Mean (UPGMA) algorithm in BioNumerics to determine the similarity between the EP, WW, CW and EN-2009 samples. The similarity matrix generated was also used for constructing a multidimensional scaling (MDS) diagram in BioNumerics. MDS is a powerful data reducing method which reduces each complex DGGE fingerprint into one point in a 3D space in a way that more similar samples are plotted closer together [19]. Additionally, EP, WW and CW DGGE bands at positions of endophytic (including chloroplast) marker bands were excised, sequenced and identified as described by Hollants *et al*. [3]. To verify their true correspondence with *Bryopsis *endophytes, excised bands' sequences were aligned and clustered with previously obtained endophytic bacterial sequences [3] using BioNumerics. Excised DGGE bands' V3 16S rRNA gene sequences were submitted to EMBL under accession numbers :HE599189-HE599213.

## Results

### Temporal stability of endophytic bacterial communities after prolonged cultivation

**Figure 1:**
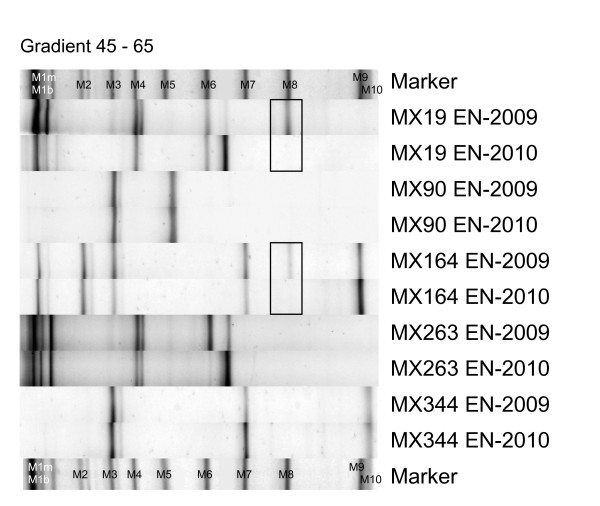
**Visual comparison of normalized endophytic DGGE fingerprints obtained from surface sterilized *Bryopsis *DNA extracts made in October 2009 (EN-2009) versus October 2010 (EN-2010)**. Differences are indicated with black boxes. The first and last lanes contain a molecular marker of which the bands correspond to known *Bryopsis *endophyte or chloroplast sequences (see additional file 2). This marker was used as a normalization and identification tool.

The endophytic bacterial communities showed little time variability after prolonged cultivation when visually comparing the normalized EN-2009 and EN-2010 DGGE fingerprints (Figure 1). The band patterns of the different MX90, MX263 and MX344 endophytic extracts were highly similar, whereas *Bryopsis *samples MX19 and 164 showed visible differences between the community profiles of their EN-2009 and EN-2010 DNA extracts. Both the MX19 and MX164 sample had lost the DGGE band representing the Phyllobacteriaceae endophytes (black boxes in Figure 1) after one year of cultivation.

### DGGE fingerprint cluster analysis: inside ≠ outside

**Figure 2:**
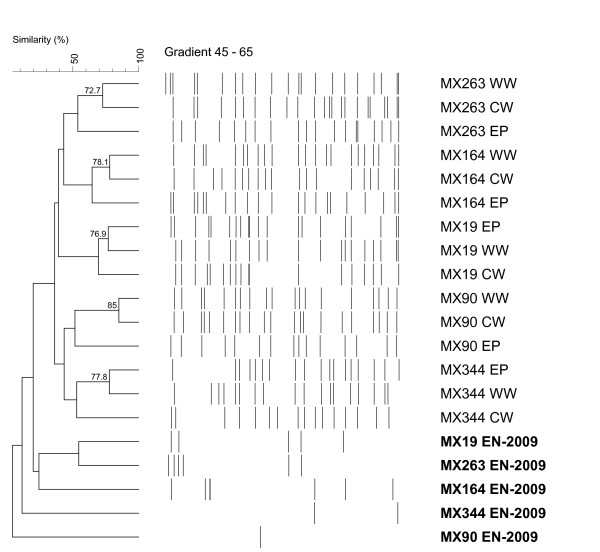
**UPGMA dendrogam showing the similarities (≥ 70%) among the endophytic (EN-2009), epiphytic (EP), washing water (WW) and cultivation water (CW) normalized DGGE fingerprints**. Cluster analysis was performed in BioNumerics using the band based Dice similarity coefficient with an optimization of 0.84% and a position tolerance of 0.48%. DGGE bands in the EN-2009 profiles identified as algal chloroplasts were excluded from the analysis. DGGE band patterns are graphically represented and similarity values above 70% are indicated above the branches.

**Figure 3:**
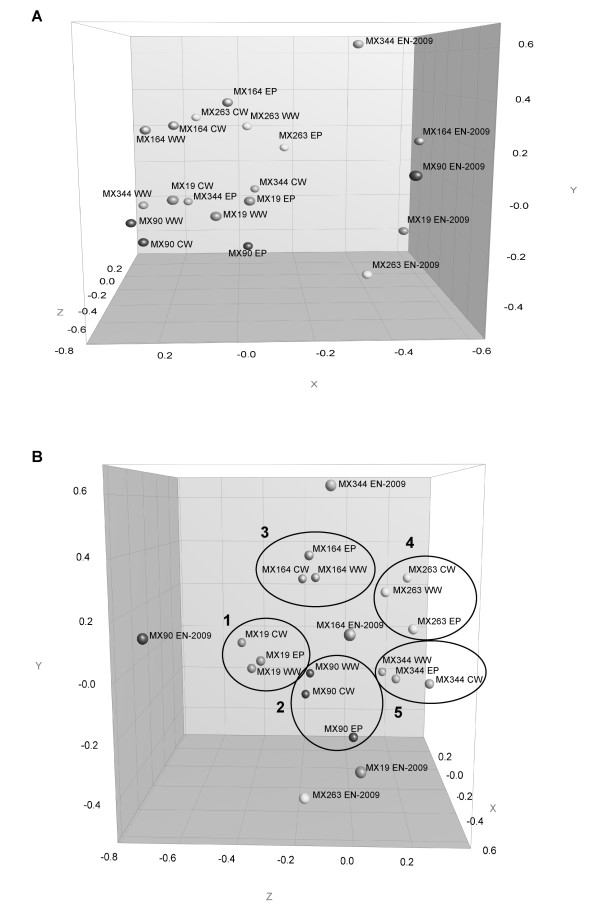
**Three-dimensional MDS plot seen from dimension X and Y (A) and Y and Z (B) visualizing the similarities among the endophytic (EN-2009), epiphytic (EP), washing water (WW) and cultivation water (CW) DGGE fingerprints**. The MDS plot was derived from the similarity matrix generated during the DGGE cluster analysis (Figure 2). Clusters 1 till 5 (B) surround the EP, WW and CW fingerprints (reduced into one point in the plot) of *Bryopsis *samples MX19, MX90, MX164, MX263 and MX344, respectively.

DGGE cluster analysis showed that the endophytic (EN) banding patterns were significantly different from the epiphytic (EP), washing water (WW) and cultivation water (CW) community profiles of all five MX *Bryopsis *cultures studied. In the dendrogram (Figure 2), the cluster containing the EP, WW and CW community profiles is clearly separated from the endophytic banding patterns (indicated in bold, Figure 2). Also the multidimensional scaling (MDS) plot (Figure 3A), which reduces the complex DGGE patterns to one point per sample, shows that the EN samples (right) are clearly apart from the epiphytic and surrounding water samples (left). Besides this, the MDS diagram showed that the EN samples did not cluster together and are distributed over the y-axis of the three-dimensional plot (Figure 3A), while the EP, WW and CW samples were more or less grouped per *Bryopsis *MX sample (Figure 3B). Within one *Bryopsis *sample EP-WW-CW cluster (clusters 1-5, Figure 3B), however, no general grouping mode can be observed. Whereas the epiphytic community samples within clusters 2, 3 and 4 (representing *Bryopsis *samples MX90, MX164 and MX263) were more apart from their corresponding WW and CW samples, this was not the case for clusters 1 and 5 (i.e. *Bryopsis *cultures MX19 and MX344). These observations corresponded to the results of the cluster analysis of all DGGE patterns (Figure 2). In addition, Figure 2 also shows a much larger diversity of DGGE bands in all epiphytic and surrounding water samples in comparison with the endophytic DGGE profiles.

### DGGE band cluster analysis: inside ≈ outside

**Figure 4:**
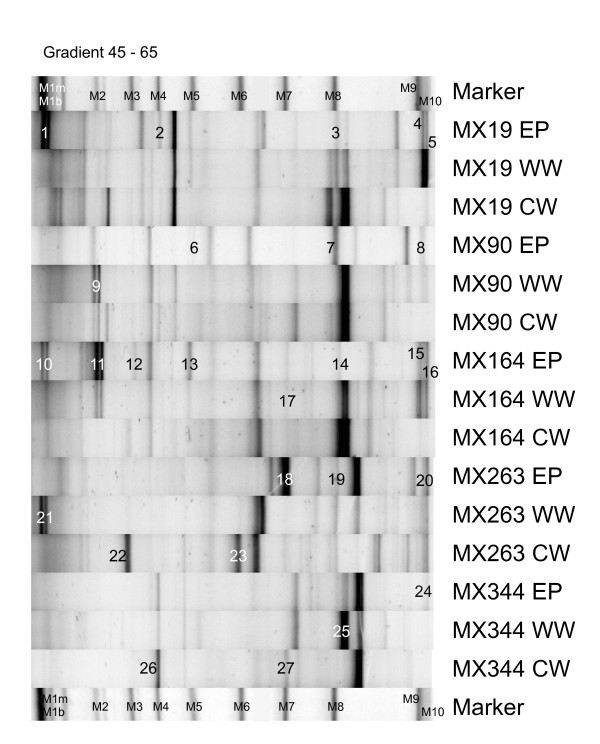
**Normalized epiphytic (EP), washing water (WW) and cultivation water (CW) DGGE fingerprints obtained from *Bryopsis *samples MX19, MX90, MX164, MX263 and MX344**. Numbers (1-27) indicate which bands were sequenced, and correspond to band numbers in Table 1 and Figure 5. The first and last lanes contain a molecular marker of which each band (M1m, M1b, M2-M10) corresponds to a known *Bryopsis *endophyte or chloroplast sequence (see additional file 2). This marker was used as a normalization and identification tool.

**Figure 5:**
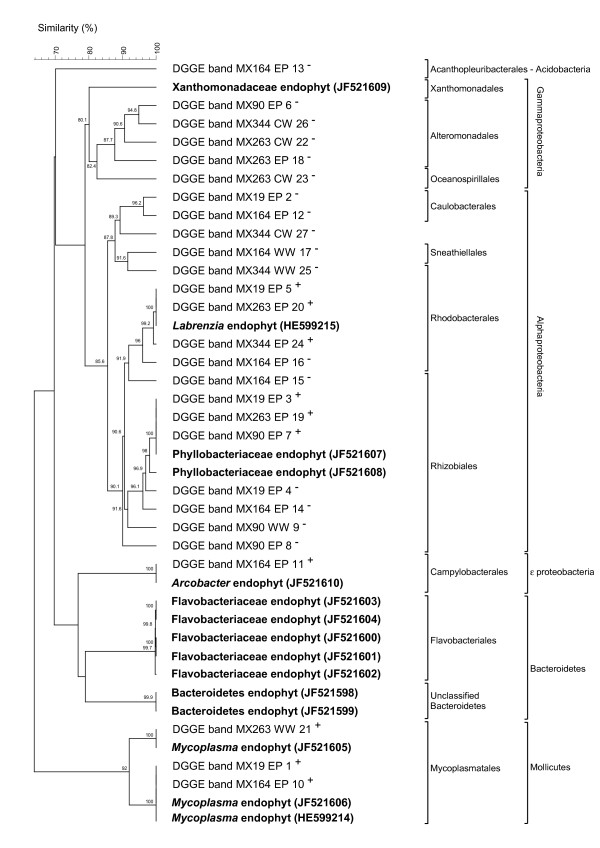
**UPGMA dendrogam showing the sequence similarities among the excised DGGE bands (numbers 1-27 in Figure 4) V3 16S rRNA gene sequences and previously obtained **[3]**endophytic bacterial full length 16S rRNA gene sequences (indicated in bold)**. Cluster analysis was performed in BioNumerics using Pearson's correlation similarity coefficients. Similarity values above 80% are given above the branches. The positive or negative correlation between the sequence identification of a certain excised DGGE band and its position towards the marker bands (see Table 1), is indicated with ^+ ^or ^-^, respectively.

Although the community fingerprints of all EP, WW and CW samples were distinct from the EN community profiles, some overlap was noticeable between individual bands from the EP, WW and CW DGGE profiles and the EN (including chloroplast) marker bands. To examine this potential overlap, EP, WW and CW DGGE bands at positions of marker bands (Figure 4, bands 1-27) were excised from the polyacrylamide gels and sequenced. Table 1 outlines the excised bands' taxonomic identification and their phylogenetic affiliation. The last column in Table 1 shows the correlation (positive^+ ^or negative^-^) between the position of a certain EP, WW or CW DGGE band towards the marker bands and its sequence identification. From this column we can deduce that most bands at positions of marker bands M1m, M2, M8 and M10 showed sequences that matched those of the marker bands and were thus identified as *Mycoplasma, Arcobacter*, Phyllobacteriaceae and *Labrenzia *species, respectively. All EP, WW or CW bands at the height of Bacteroidetes (M1b), chloroplast (M3 and M4), Flavobacteriaceae (M5-7) and Xanthomonadaceae (M9) marker bands, however, showed a mismatch. Instead of being related to *Bryopsis *endophytic bacterial sequences, these latter band sequences were affiliated with Alphaproteobacterial (Caulobacterales, Rhizobiales and Sneathiellales), Gammaproteobacterial (Alteromonadales and Oceanospirillales) and Acanthopleuribacterales sequences (see Table 1). To validate the true correspondence of excised EP, WW and CW bands with endophytic sequences, band sequences were clustered with previously obtained endophytic bacterial full length 16S rRNA gene sequences [3]. The UPGMA dendrogram (Figure 5) confirms that every one of the positively related bands (indicated with ^+^) was highly similar (≥ 99.2%) to endogenous sequences (indicated in bold). This dendrogram illustrates that *Arcobacter, Labrenzia, Mycoplasma *and Phyllobacteriaceae endogenous sequences are also present in the epiphytic, washing water and/or cultivation water bacterial communities of *Bryopsis *cultures, whereas Bacteroidetes, Flavobacteriaceae and Xanthomonadaceae sequences were strictly endogenous. In addition, *Arcobacter *and *Mycoplasma *sequences were only present in the EP, WW and/or CW bacterial communities of those *Bryopsis *MX samples in which they are also endogenously present. *Labrenzia *and Phyllobacteriaceae sequences, on the other hand, were also found in the EP, WW and/or CW bacterial communities of algal samples in which these species were not identified as being endophytic.

**Table 1:** Taxonomic identification and phylogenetic affiliation of the excised and sequenced epiphytic (EP), washing water (WW) and cultivation water (CW) DGGE bands

DGGE band number	Closest matching strain in BLAST (accession number) Query coverage/Maximum identity	Phylogenetic affiliation	Correlation
MX19 EP 1	Uncultured *Mycoplasma *sp. clone MX19.9 (JF521606) 100/100	Tenericutes; Mollicutes; Mycoplasmatales; Mycoplasmataceae	M1m + M1b -
MX19 EP 2	Uncultured bacterium clone Del10081H12 (JF262029) 100/100	Proteobacteria; Alphaproteobacteria; Caulobacterales; Hyphomonadaceae	M4 -
MX19 EP 3	Uncultured Phyllobacteriaceae bacterium clone MX19.12 (JF521607) 100/100	Proteobacteria; Alphaproteobacteria; Rhizobiales; Phyllobacteriaceae	M8 +
MX19 EP 4	Uncultured bacterium isolate TTGE gel band N68 (JN185170) 100/100	Proteobacteria; Alphaproteobacteria; Rhizobiales; Rhizobiaceae	M9 -
MX19 EP 5	Uncultured *Labrenzia *sp. clone DGGE band C (HE599215) 100/100	Proteobacteria; Alphaproteobacteria; Rhodobacterales; Rhodobacteraceae	M10 +
MX90 EP 6	Uncultured bacterium clone CD02003D03 (HM768522) 100/96	Proteobacteria; Gammaproteobacteria; Alteromonadales; Alteromonadaceae	M5 -
MX90 EP 7	Uncultured Phyllobacteriaceae bacterium clone MX19.12 (JF521607) 100/100	Proteobacteria; Alphaproteobacteria; Rhizobiales; Phyllobacteriaceae	M8 +
MX90 EP 8	Uncultured alphaproteobacterium clone TH_d327 (EU272970) 100/98	Proteobacteria; Alphaproteobacteria; Rhizobiales, Hyphomicrobiaceae	M9 -
MX90 WW 9	Uncultured bacterium clone OTU017 (GU174663) 100/100	Proteobacteria; Alphaproteobacteria; Rhizobiales; Bartonellaceae	M2 -
MX164 EP 10	Uncultured *Mycoplasma *sp. clone MX19.9 (JF521606) 100/96	Tenericutes; Mollicutes; Mycoplasmatales; Mycoplasmataceae	M1m + M1b -
MX164 EP 11	Uncultured *Arcobacter *sp. clone MX164.20 (JF521610) 100/100	Proteobacteria; Epsilonproteobacteria; Campylobacterales; Campylobacteraceae	M2 +
MX164 EP 12	Uncultured proteobacterium clone Marsh_0_33 (JF980756) 100/100	Proteobacteria; Alphaproteobacteria; Caulobacterales; Hyphomonadaceae	M3 -
MX164 EP 13	*Acanthopleuribacter pedis *type strain NBRC 101209 (AB303221) 100/93	Acidobacteria; Holophagae; Acanthopleuribacterales	M5 -
MX164 EP 14	Hyphomicrobiaceae bacterium WPS10 (HQ638980) 100/98	Proteobacteria; Alphaproteobacteria; Rhizobiales; Bartonellaceae	M8 -
MX164 EP 15	Uncultured bacterium clone I3A_12H (EU352599) 100/98	Proteobacteria; Alphaproteobacteria; Rhizobiales; Methylobacteriaceae	M9 -
MX164 EP 16	*Stappia *sp. enrichment culture clone NKiNSO2 (EU983274) 100/95	Proteobacteria; Alphaproteobacteria; Rhodobacterales; Rhodobacteraceae	M10 -
MX164 WW 17	Uncultured *Sneathiella *sp. clone w-G7 (HQ727092) 100/97	Proteobacteria; Alphaproteobacteria; Sneathiellales; Sneathiellaceae	M7 -
MX263 EP 18	*Thalassomonas *sp. UST061013-012 (EF587959) 100/100	Proteobacteria; Gammaproteobacteria; Alteromonadales; Colwelliaceae	M7 -
MX263 EP 19	Uncultured Phyllobacteriaceae bacterium clone MX19.12 (JF521607) 100/100	Proteobacteria; Alphaproteobacteria; Rhizobiales; Phyllobacteriaceae	M8 +
MX263 EP 20	Uncultured *Labrenzia *sp. clone DGGE band C (HE599215) 100/100	Proteobacteria; Alphaproteobacteria; Rhodobacterales; Rhodobacteraceae	M10 +
MX263 WW 21	Uncultured *Mycoplasma *sp. clone MX263.1 (JF521605) 100/100	Tenericutes; Mollicutes; Mycoplasmatales; Mycoplasmataceae	M1m + M1b -
MX263 CW 22	Uncultured bacterium isolate DGGE gel band B12 (HQ875697) 100/93	Proteobacteria; Gammaproteobacteria; Alteromonadales; Alteromonadaceae	M3 -
MX263 CW 23	*Alcanivorax dieselolei *strain PM07 (HM596594) 100/100	Proteobacteria; Gammaproteobacteria; Oceanospirillales; Alcanivoracaceae	M6 -
MX344 EP 24	Uncultured *Labrenzia *sp. clone DGGE band C (HE599215) 100/100	Proteobacteria; Alphaproteobacteria; Rhodobacterales; Rhodobacteraceae	M10 +
MX344 WW 25	*Ruegeria mobilis *strain F4122 (HQ338148) 100/99	Proteobacteria; Alphaproteobacteria; Rhodobacterales; Rhodobacteraceae	M8 -
MX344 CW 26	Uncultured bacterium clone EMar8 (FR667032) 100/94	Proteobacteria; Gammaproteobacteria; Alteromonadales	M4 -
MX344 CW 27	Uncultured bacterium clone W2-97 (HQ322761) 100/90	Proteobacteria; Alphaproteobacteria	M7 -

The band numbers correspond to the numbers (1-27) in Figure 4. The last column shows the correlation (positive ^+ ^or negative ^-^) between the identification of a band and the sequence information of the marker band (M1m, M1b, M2-M10) at the same position.

## Discussion

The existence of highly specific macroalgal-bacterial associations is no longer doubted [7]. Various studies revealed that bacterial communities living on macroalgae clearly differ from those occurring in the surrounding seawater [4,5,8,20]. These studies, however, focused on the distinctiveness of the epiphytic bacterial communities from the free-living environmental communities and never studied the specificity of the endophytic bacteria associated with macroalgae. To our knowledge, this is the first study to address the temporal variability of the endogenous (EN) bacterial communities of *Bryopsis *isolates and their distinctiveness from the epiphytic (EP) and surrounding water (WW and CW) bacterial communities after prolonged cultivation using the DGGE technique. Taken the inherent limitations of the DGGE technique into account [21], we observed that the endophytic bacterial community profiles were notably different from the fingerprints of bacterial communities on and surrounding *Bryopsis *cultures. DGGE fingerprint cluster analysis (Figure 2) and MDS (Figure 3) clearly indicate that the epiphytic and surrounding water samples in all *Bryopsis *cultures were more similar to each other than to their corresponding endophytic community profile. This suggests the existence of specialized endophytic bacterial communities within *Bryopsis *algae which are clearly distinct from the outer surface and environmental bacterial communities. This apparent specificity is supported by the observation that *Bryopsis *harbors rather stable endophytic bacterial communities, which showed little time variability after one year cultivation of the algal samples (Figure 1). However, examination of individual DGGE bands did reveal some similarities between intra- and extracellular bacteria. While Bacteroidetes, Flavobacteriaceae and Xanthomonadaceae species seemed exclusively endobiotic, sequence cluster analysis confirmed that *Arcobacter, Labrenzia, Mycoplasma *and Phyllobacteriaceae endophytes were also present in the epiphytic, washing water and/or cultivation water extracts. This latter observation is consistent with the outcome of a study conducted by Maki *et al*. [22] which revealed similar intracellular and extracellular bacterial populations in and on the harmful marine microalga *Heterocapsa circularisquama *in culture.

Although the *Bryopsis *cultures used in this study have been kept in the laboratory for almost three years due to experimental restrictions [3], our data allow us to put forward some hypotheses regarding the nature of the endophytic communities within natural *Bryopsis *populations. Whereas we cannot rule out selection by artificial laboratory growth conditions, *Arcobacter, Labrenzia, Mycoplasma *and Phyllobacteriaceae endophytes can at least survive without the *Bryopsis *host, suggesting they might be facultative endogenous bacteria which are acquired from the local environment. This is consistent with the general perception that most plant endophytes originate from the surrounding environment and the outer plant surface [23,24]. Bacteroidetes, Flavobacteriaceae and Xanthomonadaceae endophytes, on the other hand, appear well adapted to an endobiotic lifestyle as they persist within the *Bryopsis *interior after prolonged cultivation. Especially Flavobacteriaceae endophytes, which are present in all five MX samples collected hundreds of kilometres apart, might be obligate endophytes which are strictly dependent on the *Bryopsis *host for their growth and survival. This co-occurrence of multiple facultative and obligate bacterial endophytes is also well documented in many land plant and insect hosts [23,25].

Furthermore, the *Bryopsis *endophytic communities seem also rather specific as the EP, WW and CW extracts contained numerous Alphaproteobacterial, Gammaproteobacterial and Acanthopleuribacterales species which are not present in the EN samples. This apparent specificity is confirmed by our observations that EP, WW, CW (data not shown) and EN (see Figure 1) extracts made at different time points revealed largely consistent banding patterns even after the algal specimens were repeatedly wounded and transferred to fresh, sterile cultivation medium (see material and methods section). Consequently, the *Bryopsis *host seems able to selectively maintain its endophytic flora and/or to attract specific facultative endophytes after wounding. Although this may be the result of more general physiological and biochemical processes [7], the characteristic properties of *Bryopsis *might also contribute to this selectiveness. An interesting characteristic of *Bryopsis *is that following cell wounding, the protoplasm can aggregate and regenerate into a mature individual. This process involves a transient state of membrane-free protoplasts in seawater [13]. Although this transient 'life without a membrane' state might seem anything but selective, Klotchkova and coworkers [26] showed that an incompatibility barrier is present during protoplast formation to exclude foreign inorganic particles or alien cell components. Only some chosen cells or particles could be incorporated into *Bryopsis *protoplasts. Moreover, the lectins which play a key role in the aggregation process during protoplast formation [27-30] might actually be 'specificity mediators'. The description of the *Bryopsis *specific lectin Bryohealin by Kim *et al*. [29], which contains an antibiotic domain that protects the newly generated protoplasts from bacterial contamination [30], supports this hypothesis. Lectins are known symbiosis mediators in, for example, legume-rhizobia and sponge-bacterial symbioses [31,32].

Besides the endophytic bacterial communities, also the epiphytic and the surrounding cultivation water bacterial communities seemed unique to each *Bryopsis *culture as the EP, WW and CW fingerprints of a given *Bryopsis *sample clearly clustered together. This is consistent with the general perception of highly specific macroalgal-bacterial interactions as discussed above [7]. Additionally, since all five *Bryopsis *cultures were maintained under similar laboratory conditions, the above observation suggests that factors other than cultivation conditions contributed to the observed specificity (see Material and methods section).

## Conclusion

Our results indicate that *Bryopsis *samples harbor specific and rather stable endophytic bacterial communities after prolonged cultivation which are clearly distinct from the epiphytic and surrounding cultivation water bacterial communities. Even though *Bryopsis *algae are repeatedly being exposed to a mix of marine bacteria, they seem to selectively maintain and/or attract their endophytes after repeated wounding events in culture. Despite the limitations of the experimental design, this indicates that *Bryopsis *has some intrinsic mechanisms to favour the entry of certain bacteria of possible ecological importance within its cell, suggesting macroalgal- bacterial endobioses might be as or even more specific than macroalgal-epiphytic bacterial associations. The use of species-specific primers and probes may open the way to investigate the specificity, both spatially and temporally, of the endophytic communities in natural *Bryopsis *populations.

## Authors' contributions

JH designed the experiments, analysed the data and wrote the paper. FL maintained the algal cultures. JH and HD performed the experiments. FL, ODC and AW conceived the study and helped to draft the manuscript. All authors read and approved the final manuscript.

## Supplementary Material

Additional file 1 **Transmission electron micrograph of vegetative *Bryopsis *thallus in longisection**. Figure A: the outer cytoplasmic layer (ol) adjacent to the *Bryopsis *cell wall (cw) contains most of the organelles excluding only the chloroplasts (chl), which are present in the inner layer next to the central vacuole (cv). Magnification: × 8000, Scale bar: 3 μm. Figure B (detail of Figure A): besides mitochondria (m), endoplasmic reticulum and vacuolar evaginations (v), endogenous bacteria (ba) are present in the outer cytoplasmic layer. Magnification: × 25000, Scale bar: 1 μm. Click here for file

Additional file 2 **The marker used as a normalization and identification tool in all DGGE analyses**. This marker covers the full range of endophytic (including chloroplast) sequences previously obtained from *Bryopsis *samples MX19, MX90, MX164, MX263 and MX344 [3]. For each marker band, the band name (M1m, M1b, M2-M10), taxonomic identification, clone reference and accession number are represented. Click here for file
