# Supplementary figures and images for: Life without a cell membrane: Challenging the specificity of bacterial endophytes within Bryopsis (Bryopsidales, Chlorophyta)
Source: BMC Microbiol. 2011 Nov 21;11:255. doi: 10.1186/1471-2180-11-255 (PMC3252328; doi:10.1186/1471-2180-11-255)

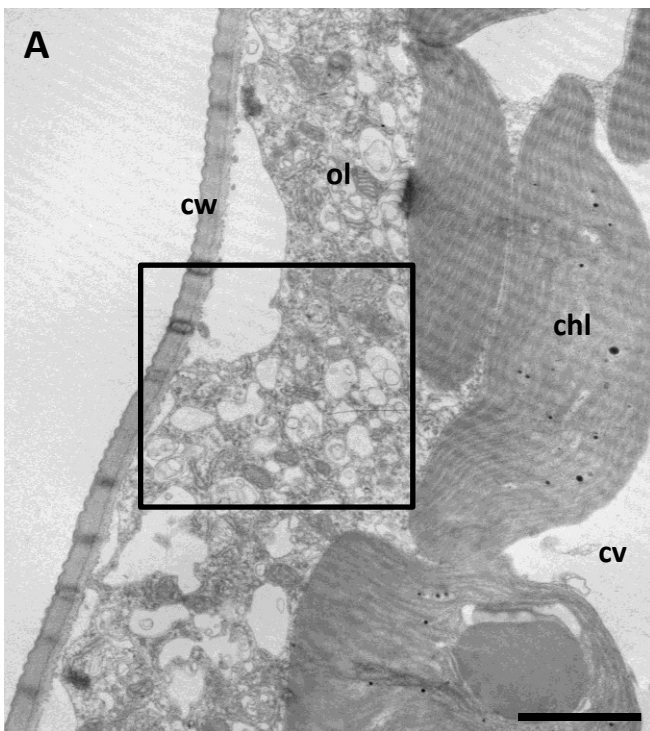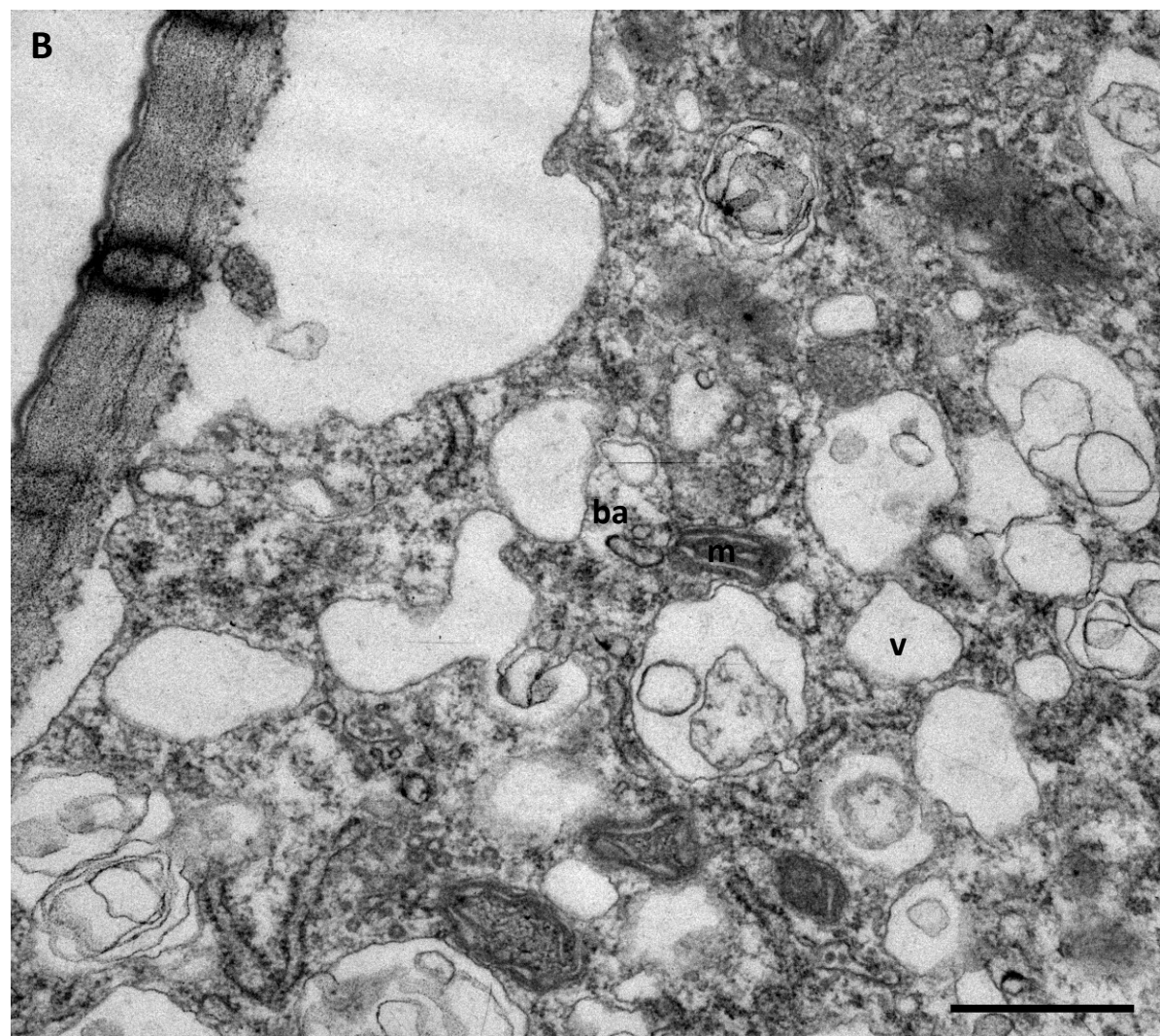

Supplement: Additional file 1 — Transmission electron micrograph of vegetative Bryopsis thallus in longisection. Figure A: the outer cytoplasmic layer (ol) adjacent to the Bryopsis cell wall (cw) contains most of the organelles excluding only the chloroplasts (chl), which are present in the inner layer next to the central vacuole (cv). Magnification: × 8000, Scale bar: 3 μm. Figure B (detail of Figure A): besides mitochondria (m), endoplasmic reticulum and vacuolar evaginations (v), endogenous bacteria (ba) are present in the outer cytoplasmic layer. Magnification: × 25000, Scale bar: 1 μm. [file 1471-2180-11-255-S1.PDF]
