# Supplementary material for: Life without a cell membrane: Challenging the specificity of bacterial endophytes within Bryopsis (Bryopsidales, Chlorophyta)
Source: BMC Microbiol. 2011 Nov 21;11:255. doi: 10.1186/1471-2180-11-255 (PMC3252328; doi:10.1186/1471-2180-11-255)
Supplement: Additional file 2 — The marker used as a normalization and identification tool in all DGGE analyses. This marker covers the full range of endophytic (including chloroplast) sequences previously obtained from Bryopsis samples MX19, MX90, MX164, MX263 and MX344 [3]. For each marker band, the band name (M1m, M1b, M2-M10), taxonomic identification, clone reference and accession number are represented. [file 1471-2180-11-255-S2.PDF]

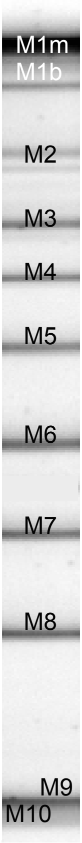

M1m, M1b	*Mycoplasma* & Bacteroidetes endophytes	clone MX19.8 (JF521598)<br>clone MX19.9 (JF521606)<br>clone MX263.1 (JF521605)<br>clone MX263.73 (JF521599)
M2	*Arcobacter* endophyte	clone MX164.20 (JF521610)
M3	*Bryopsis* chloroplast	clone MX90.9 (JF521615)
M4	*Bryopsis* chloroplast	clone M19.1 (JF521612)
M5	Flavobacteriaceae endophyte	clone M90.40 (JF521602)
M6	Flavobacteriaceae endophyte	clone M263.61 (JF521604)
M7	Flavobacteriaceae endophyte	clone M344.2 (JF521601)
M8	Phyllobacteriaceae endophyte	clone M19.12 (JF521607)
M9	Xanthomonadaceae endophyte	clone M164.9 (JF521609)
M10	*Labrenzia* endophyte	DGGE band C (HE599215)
